# Multi-Element Profile Characterization of Monofloral and Polyfloral Honey from Latvia

**DOI:** 10.3390/foods12224091

**Published:** 2023-11-11

**Authors:** Kriss Davids Labsvards, Vita Rudovica, Anastasija Borisova, Kristina Kokina, Maris Bertins, Jevgenija Naumenko, Arturs Viksna

**Affiliations:** 1 Department of Chemistry, University of Latvia, Jelgavas Street 1, LV-1004 Riga, Latvia; vita.rudovica@lu.lv (V.R.); maris.bertins@lu.lv (M.B.); jevgenija2308@gmail.com (J.N.); arturs.viksna@lu.lv (A.V.); 2 Institute of Food Safety, Animal Health and Environment “BIOR”, Lejupes Street 3, LV-1076 Riga, Latvia; anastasija.borisova@bior.lv (A.B.); kristina.kokina@bior.lv (K.K.)

**Keywords:** honey, floral origins, inductively coupled plasma mass spectrometry, element profile, proteins, chemometrics

## Abstract

Honey is of scientific interest mainly due to its health-promoting and antibacterial properties, which are also associated with its floral origins. However, the methods for confirming honey floral origins are quite limited and require improvements. One method suggested in the search for a multi-method approach to evaluating the floral origins of Latvian honey is inductively coupled plasma mass spectrometry (ICP-MS). This study investigated the multi-element profile of 83 honey samples of well-specified floral origins. The main findings included using Ba, Ca, Cs, Fe, and Rb as indicator elements for heather honey. The chemometric evaluation supported the use of ICP-MS for distinguishing heather honey from other types of honey. The Latvian polyfloral honey multi-element profile was defined and compared to honey samples with other geographical origins. Additionally, the multi-element profiles of buckwheat, clover, and polyfloral honey proteins were investigated to clarify whether the majority of elements were bound with proteins or not. Preliminary results indicated that Ca, K, Mg, Mn, Na, and Sr were mainly found in non-protein-bound forms, while the majority of Al, Cu, Ni, and Zn were in the form of large chemical structures (>10 kDa).

## 1. Introduction

Honey is a natural sweetener, which is well regarded for its taste [1] and for its health-promoting [2] and antibacterial properties [3]. Floral origins play a major role in its organoleptic properties such as taste [4,5], as well as its health benefits [6,7]. Therefore, determining its floral origins is crucial to guaranteeing the correct labeling of honey used as a food product [8].

Currently, melissopalynology analysis is the primary method of floral origin confirmation. However, the method has significant drawbacks that have not yet been managed. Some plants, like citrus or lavender [9], reproduce asexually, and are therefore unable to produce pollen. The need for specifically qualified personnel [10] could be resolved with artificial-intelligence (AI)-generated pollen recognition software, but this requires specific tools and hardware and is limited by database selection [11]. The validation of uncommon plant species origins of honey is quite challenging since plant pollen is usually overrepresented or underrepresented in honey, thus complicating the judgment of whether honey is monofloral or not [12].

Therefore, the following modern instrumental methods [13] available in common chemistry laboratories are often chosen as alternatives for evaluating honey floral origins: headspace solid-phase microextraction/gas chromatography–mass spectrometry (HS-SPME/GC–MS) [14,15], ultra-high-performance liquid chromatography–electrospray ionization–tandem mass spectrometry (UPLC–ESI–MS/MS) [16], nuclear magnetic resonance (NMR) [17], Fourier transformation infrared spectroscopy with the attenuated total reflectance sampling technique (FTIR-ATR) [18], isotope ratio mass spectrometry (IRMS) [19], Raman spectroscopy [20], inductively coupled plasma optical emission spectrometry (ICP-OES) [21], and inductively coupled plasma mass spectrometry (ICP-MS) [22]. Most of these methodologies are novel and are not implemented in laboratories as daily routine analyses [23]. One of the exceptions is honey macro- and micro-element determination, since honey is considered rich in nutrients and is an in-demand product for analysis to provide the correct labeling [24,25].

ICP-MS and ICP-OES are well regarded as the most common methods for determining total element concentration in various food matrices [26,27,28], including honey [29]. A crucial step before analysis is sample preparation, which for the ICP-MS methodology includes crushing, extraction, homogenization, lyophilization, and washing, with sample digestion being considered the most crucial [30]. Among the various digestion procedures, digestion in acidic conditions (HNO_3_/H_2_O_2_) using a microwave oven is the most preferred [31].

This study’s main aim was to complement the development of a strategy to enable Latvian beekeepers to confirm the floral origins of their honey using modern analysis methods. Recent studies have investigated applications of C and N isotope ratios using IRMS, a polyphenol profile using UPLC-HRMS, and aliphatic and polyphenol compound profiles using NMR [32]. Multi-element profile characterization using ICP-MS would be the perfect improvement for an all-around instrumental strategy. Within the scope of this study, there was a need to determine which elements were the most characteristic of particular floral origins. It is important to define local polyfloral product composition, so the information in this study can be useful for international comparisons, as well as for international advertising by entrepreneurs. Finally, the multi-element profiles of honey and its proteins were compared to give better insight into possible element sources and bioavailability, as well as to critically evaluate the method’s overall success.

## 2. Materials and Methods

### 2.1. Samples

**Figure 1:**
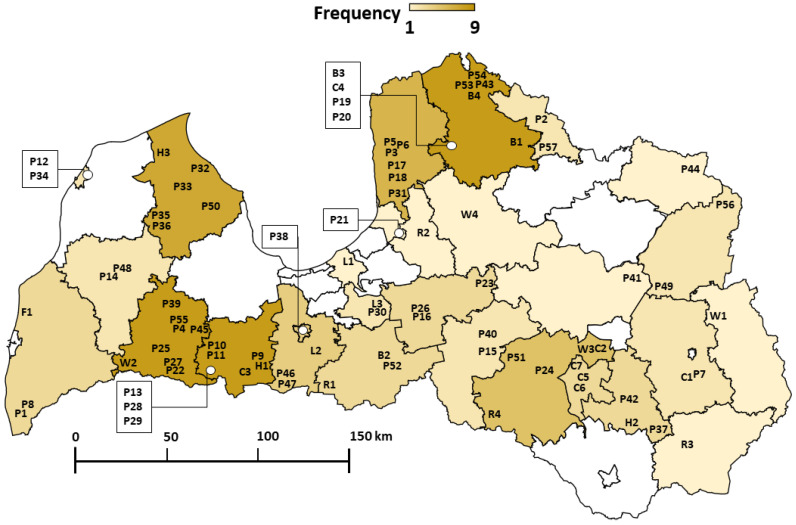
Locations of collected buckwheat (B), clover (C), heather (H), linden (L), rapeseed (R), and willow (W) monofloral honey samples and polyfloral (P) honey samples. Sample frequency color code within districts is described in the legend.

A total of 83 honey samples were collected from 2019 through 2021 in Latvia. All samples were reported as natural and gathered from local beekeepers. Sample locations are summarized in Figure 1.

The pollen composition was studied using melissopalynology analysis, identifying 500 grains per sample [33]. The 26 samples fulfilled the criteria for monofloral honey from 8 different monofloral classes. The information about the dominant pollen composition of monofloral honey is summarized in Table 1.

### 2.2. Total Element Determination in Honey with ICP-MS

#### 2.2.1. Sample Preparation with Two-Stage Mineralization

The honey samples were mineralized using a previously published method [35] and modified for multi-element determination in a high-sugar-content matrix. A 1.0 ± 0.3 g honey sample was weighed in a PTFE digestion vessel. The sample was diluted with 4 mL ultra purity water (resistance ~18 MΩ·cm), 5 mL 69% HNO_3_, and 1 mL 30% H_2_O_2_. The container with the sample and reagent mixture was placed in a fume hood for at least 20 min to complete the reaction. After that, the vessel was capped and placed in a Mars 6 microwave oven (CEM Corporations, Matthews, NC, USA). The first stage of mineralization was as follows: The sample was heated to 70 °C within 20 min and held for 5 min, then the temperature was raised to 100 °C within 20 min and held for 10 min. At the end of the first step, the sample was heated to 120 °C within 15 min and held for 10 min. Then, the sample was cooled to a temperature of ≤50 °C and carefully opened in the fume hood to release pressure. The second stage of mineralization continued as follows: the sample was heated to 120 °C within 30 min and held for 10 min, and finally the temperature was raised to 170 °C within 15 min and held for 15 min. In both stages, the microwave oven was set to 1300 W. The sample was cooled again to ≤50 °C and transferred with 0.5% HCl solution to a 50 mL volumetric flask. Pt was added as an internal standard (Fluka, 1000 mg/L) to obtain a final concentration of 5 μg/L. The sample solution was transferred to a PPE tube and stored in a refrigerator at a +5 °C temperature until instrumental analysis.

#### 2.2.2. Instrumental Settings

For multi-element total concentration determination, the ICP-MS Agilent 7700× (Agilent technologies, Santa Clara, CA, USA) was used. The optimal instrumental settings are summarized in Table 2. For validation purposes, we used the Mercury standard 10 μg/mL (LGC, Manchester, NH, USA) and the “multi-element standard solution 5 for ICP”. The following concentrations were used: 100 mg/L—Ca, Fe, K, and Na; 10 mg/L—Ag, Al, Ba, Be, Bi, Cd, Co, Cr, Cs, Cu, Ga, In, Li, Mg, Mn, Mo, Ni, Pb, Rb, Sr, Tl, V, and Zn (Sigma-Aldrich, St. Luis, MO, USA).

### 2.3. Element Determination in Honey Proteins with ICP-TQMS

#### 2.3.1. Sample Preparation of the Dilute and Shoot Method

Honey proteins were purified using the dialysis method from previous stable carbon and nitrogen isotope studies [33]. The approximately 12.0 ± 8.0 mg protein sample was weighed in a 15 mL PPE tube, diluted with 4 mL ultra purity water and 4 mL 69% HNO_3_, and treated with an ultrasound bath until the solution was clear.

#### 2.3.2. Instrumental Settings

The multi-element profile of honey proteins was determined using the inductively coupled plasma triple-quadrupole mass spectrometer (ICP-TQMS) Agilent 8900 Triple Quadrupole (Agilent Technologies, Santa Clara, CA, USA) instrument in MS/MS mode. The instrumental settings for protein element determination are summarized in Table 3.

For protein screening purposes, the “68 Component ICP-MS standard at 10 µg/mL” (High-Purity Standards, Charleston, SC, USA) was used and diluted within a range of 1 to 500 μg/L for calibration graphs and the determination of multi-element concentrations in proteins.

### 2.4. Software and Statistical Analysis

For element determination in honey, the MassHunter Workstation vB.01.03 software was used (Agilent Technologies, Santa Clara, CA, USA). Analysis of variance (ANOVA), principal component analysis (PCA), hierarchical cluster analysis (HCA), and Dixon r10 outliner were performed using Minitab 17 Statistical software (Minitab, Brandon Court, UK). One-way ANOVA Fisher comparison tests were performed using a 95% confidence level for statistically significant difference confirmation. PC score values and loadings were calculated from the correlation matrix. Outliers from the PCA were obtained using the Mahalanobis distance evaluation. HCA was performed with standardized variables, the Wards method as a linkage method, and the Euclidean distance as a measure. Dixon’s r10 test was performed for descriptive statistics.

## 3. Results and Discussion

### 3.1. Validation of the ICP-MS Method for High-Carbohydrate Samples

To attain favorable lower detection limits, a two-step mineralization process was employed, followed by validation with honey to ensure the accuracy of the results. The summarized validation results are presented in Table 4.

For the 30 different elements, the limits of detection (LODs) ranged from 0.0002 to 0.75 mg/kg, while the limit of quantification (LOQ) values ranged from 0.0005 to 2.5 mg/kg. The LOQ for each element was set as the lowest calibration point. Most elements demonstrated excellent linearity (R^2^ ≥ 0.9998), with the exception of mercury (Hg) at R^2^ = 0.992. The recovery fell within the acceptable range of ± 20% (85–116%), and the repeatability did not exceed the 10% relative standard deviation (RSD). Based on these validation results, the method is suitable for multi-element characterization in honey.

### 3.2. Element Concentrations in Honey by Its Floral Origins

The amount of the above 30 elements in Latvian honey was analyzed, thereby giving a broad set of results (see Supplementary Materials Tables S1–S5). Table 5 summarizes the 12 elements found in most samples over the LOQ and reveals information for distinguishing potential floral origins.

The preliminary results showed that a multi-element chemical profile can be used to distinguish heather honey from other monoflorals or polyfloral honey. The nutrient elements Ca and Fe in heater honey showed significant differences from the other floral origins in this study. Ca and Fe are usually monitored for nutrient-labeling purposes, but the current results can also help the apiculture sector to evaluate the presence of possible monofloral heather honey. Heather is acknowledged as a metallophyte, i.e., a genetically metal-tolerant plant. The results in Table 5 indicate significant differences in the concentration of the metals Rb, Cs, and Ba between heather and other floral origins.

In the heather honey samples, the estimated As concentration was near the LOQ. Lottermoser et al. [36] reported that heather tends to accumulate As; therefore, the plant itself is As-enriched. That finding is in good agreement with this study, as heather honey showed significant As accumulation. Although As might seem a selective indicator of heather floral origins, it must be thoroughly investigated, since As was also found in three other honey samples with polyfloral origins (P1, P5, P15) with no Ericaceae pollen reported. Because of low occurrence, all were excluded as outliers (see Tables S2 and S3). It must be noted that As-containing samples do not share common locations and are scattered throughout the entire country. Rb is known to compete with the micro-element K for the same uptake properties in plants due to similar properties. Usually, low K in soil is compensated for by Rb [37,38]. The K content in heather honey was not significantly different from the other studied floral origins but tended to be relatively high, while in the case of Rb, it was exceptionally high. ^133^Cs is considered to be efficiently taken up by rapeseed [39], but our study showed no accumulation in honey. Otherwise, it was quantified in heather honey (0.24 ± 0.9 mg/kg) and found in polyfloral honeys rich with heather pollen (P2, P3, P8). Ba is the 14th most abundant element on Earth, and its concentration in soil usually ranges from 19 to 2300 mg/kg [40], generally causing stress to plants [41]. In the Latvian monofloral honey samples, it was significantly higher in heather honey. Therefore, floral origins must be considered when honey is used for monitoring environmental pollutants [42]. Heather honey showed a significant Tl concentration (0.030 ± 0.002 mg/kg), which is a highly toxic heavy metal [43] with the ability to mimic K in biochemical processes [44].

It is important to define the local typical polyfloral honey characteristics when applying methods for distinguishing honey floral origins. The current data show only significant differences for heather honey distinction, but deviations from polyfloral signature concentrations could be useful indicators. The task is complicated because of the variety of polyfloral sample characteristics (see Table 5), which are described with large standard deviations. Polyfloral honey is a mixture of honey gathered from different kinds of plants and different kinds of territories; therefore, nearly every sample is unique in its composition and a large standard deviation is common.

Al was found >LOQ in the majority of samples, except for honey of linden floral origins. The concentration range was from 0.83 to 5.22 mg/kg. Co was found altogether in 18 samples and the results showed a mean value 2/3 of the LOQ value, confirming Co presence in honey but lacking the correct quantified concentration. Similarly, Ni was found >LOD in 36 samples and a mean was slightly over the LOD. Zn was quantified in the majority of the samples of different floral origins, except linden honey. The concentrations ranged from 0.29 to 6.48 mg/kg, and differences between monofloral groups were not statistically significant. The heavy metal Pb was found in 21 polyfloral honey samples and was not associated with any floral type. 

Although only heather honey showed significant differences from the other floral groups, the element chemical profile of the listed elements in Table 5 can be used for floral source approximation or determination using signature concentration evaluation. The majority of Ni, Zn, and Pb concentrations in monofloral groups were below the LOQ, making it difficult to distinguish among polyflorals or other monofloral groups. Nine elements (Li, Be, Cr, Se, Mo, Ag, Cd, Sb, and Hg) were found at least once in the Latvian honey, and two elements (V, Ga) were not found at all and reported below the LOD. Cr was found in samples F1 (0.054 mg/kg) and P41 (0.072 mg/kg). Although F1 is a monofloral Phacelia honey, there was no *Phacelia tanacetifolia* pollen in P41; thus, Cr was not associated with Phacelia honey.

Attention must be paid to certain elements like Cr, Co, and Cs. These elements, in the majority of samples, had concentrations below the LOD. There are several substitution methods found in the literature using the LOD, LOD/√2, and 0. In the authors’ opinion, substituting values below the LOD with LOD/√2 may be the most appropriate and arguably the safest substitution method because the LOD is the upper limit and can significantly increase mean values, while 0 is the lowest possible limit and will definitively decrease the mean values found in nature if the LOD is not a limiting factor. The choice of /√2 is based on the assumption that data below these limits follow a “triangular distribution” [45]. Value substitution is not always favorably regarded or recommended [46] and could sometimes be considered as meddling with data. Therefore, the simple removal of one value from the resulting pool (described as an outlier value) is the most popular choice [47]. Although it gives better insights in general, it could be misleading when 60% of the data are below the LOD. No data below the LOD were substituted in this publication, but the total Cr, Co, and Cs concentrations should be carefully assessed. The main “cure” for this issue would be enhancements in the sensitivity of the method. The triple-quadrupole instrumentation could be a way to improve sensitivity [48], but it is not common in laboratories.

### 3.3. Polyfloral Honey Element Chemical Profile

It is essential to define national food resource characteristics. First, we must be aware of whether the composition comes with health benefits [49] or risks [50]. Second, product screening at the national level is significant if the characteristics are outstanding in the international context; put simply—if the food is outstanding.

**Figure 2:**
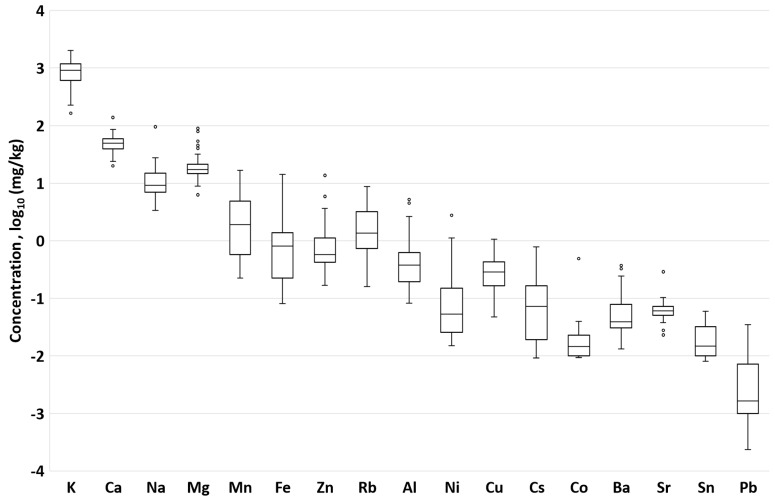
Boxplot of the 17 most abundant macro- and trace element concentrations (mg/kg) in Latvian polyfloral honey expressed as a logarithm of base 10. The outliers are depicted with white circle.

To define the polyfloral chemical profile of honey gathered by bees in Latvia, the concentrations of elements are summarized in the boxplot in Figure 2. Sample locations were spread throughout the entire country, representing coastal, urban, plain, forest, and upland areas. The 17 elements ranked in order of average concentrations are K > Ca > Mg > Na > Mn > Rb > Fe > Zn > Al > Cu > Cs > Ni > Ba > Sr > Sn > Co > Pb.

The logarithmic values of the estimated macro-element and trace element concentrations are summarized in the boxplot in Figure 2. The boxplot contains only the elements found in at least 16 samples over the LOD.

The K concentration (900 ± 500 mg/kg) was notably higher than other determined elements, which complied with other studies [51,52], but even honey with a relatively high potassium concentration is not a notable K source, since the portion requirements are >200 mg [53].

Latvian polyfloral honey K concentration:

- is higher than reports of Ethiopian [54] and Serbian honey [21];
- could be considered the same—but with higher means—as Estonian [55], Yemenian [56], Moroccan [57], Argentinian [58], Brazilian, and Portuguese honey [59];
- could be considered the same—but with lower means—as Polish [60,61,62], Slovenian flower [63], Hungarian linden [64], Romanian linden [65], Bulgarian linden [66], Italian [67], Malaysian [68], and New Zealand honey [69];
- is lower than reports of Hungarian meadow sage [70] and Tunisian wildflower honey [71].

The concentrations of the other determined macro-elements Ca (51 ± 18 mg/kg), Mg (22 ± 15 mg/kg), and Na (13 ± 13 mg/kg) were in good agreement with other studies [64].

Trace elements: Compared with Tunisian wildflower honey concentrations, certain elements—Mn, Ni, Cu, Zn, and Cr—were in good agreement, but Pb concentrations in Latvian honey were 80-fold lower. The Se in the two samples P15 and P49 met the concentration range in Tunisian wildflower honey: 0.058 and 0.040 mg/kg, respectively. In other studies, when compared with geographically closer countries, the trace element concentrations were similar [60].

A wide range of estimated element content in polyflorals was shown for Fe (0.3–14 mg/kg), Pb (0.0005–0.04 mg/kg), and Cs (0.009–0.8 mg/kg). The polyfloral samples containing relatively high heather pollen content but still not exceeding the necessary 25% pollen percentage limit showed relatively high Cs concentrations—P8 (0.78 mg/kg), P15 (0.14 mg/kg), and P20 (0.15 mg/kg)—thus inflating the polyfloral characteristic range.

Since polyfloral honey is a mixture of various floral origins, it was predictable that outlying samples occurred during the element chemical profile evaluation. In the case of Al, the highest concentrations were found in the P48 and P50 samples. Both had relatively high raspberry pollen percentages: 25% and 37%, respectively. W1, P56, and P57 were outliers for Fe with 2.3, 14.3, and 4.4 mg/kg, respectively. Compared to other studies, the concentrations seemed regular and are not necessarily associated with possible anthropogenic contamination [72,73,74], and this supplements previous studies of Latvian honey [75].

The elements withdrawn from the boxplot summary were Ag, As, Be, Cd, Cr, Ga, Hg, Li, Mo, Sb, Se, Tl, and V, since those elements were found in fewer than 16 samples and are not considered notably representative of Latvian polyfloral honey. As, however, can be used for monofloral Latvian honey evaluation. Even though there is no application or evidence for discrimination in the most common floral origins of Latvia, the monitoring of these elements in honey is recommended. Bees can gather honey from plants within an approximate area of 7 km^2^; therefore, it can be applicable for environmental monitoring purposes [76].

### 3.4. Comparison of Honey Element Composition and Pollen Percentage

Melissopalynology analysis identified 22 different plant pollens in Latvian honey of various origins. The correlation coefficients calculated using Pearson’s method between element concentrations and pollen percentages are presented in Supplementary Information Figure S1.

According to Evans’ interpretation [77], a range of positive correlations, ranging from “very strong” to “very weak,” was observed between pollen types and element concentrations. Specifically, two “very strong” correlations (0.8 ≤ r < 1.0) were identified: one between broad bean (*Vicia faba*) pollen and Tl concentration in honey (r = 0.909), and another between broad bean pollen and Cs concentration (r = 0.836).

Additionally, six “strong” correlations (0.6 ≤ r < 0.8) were found, including rapeseed (*Brassicaceae*) pollen with Se (r = 0.636) and Tl (r = 0.615) concentrations, umbellifer (*Apiaceae*) pollen with Cd concentration (r = 0.763), buckwheat (*Fagopyrum esculentum*) pollen with Se concentration (r = 0.745), and heather (*Ericaceae*) pollen with Ba concentration (r = 0.673). Furthermore, meadowsweet (*Filipendula ulmaria*) pollen, though occasionally found in honey, exhibited a “strong” correlation with Pb concentration (r = 0.600).

Notably, no “very strong” to “strong” negative correlations were detected, as was expected for this dataset. These correlation results complement the significant differences observed in Ba concentrations in heather honey compared to other floral groups. Fe concentration also exhibited a significant difference, with a “moderate” correlation with heather pollen (r = 0.579). Although Ca, Cs, and Rb concentrations were significantly different, their correlation coefficients varied from “weak” positive to “weak” negative.

### 3.5. Multi-Element Concentrations in Honey Proteins

Proteins in honey serve as valuable nutrition sources, primarily fulfilling the role of nourishing bee larvae [78]. These proteins contain various metals integrated into their structures and are not readily available as free ions. In previous studies [33], an examination of protein C and N content and their isotope values was conducted. The proteins were purified using the dialysis method, ensuring their freedom from sugars, other small molecular compounds, and ions.

**Figure 3:**
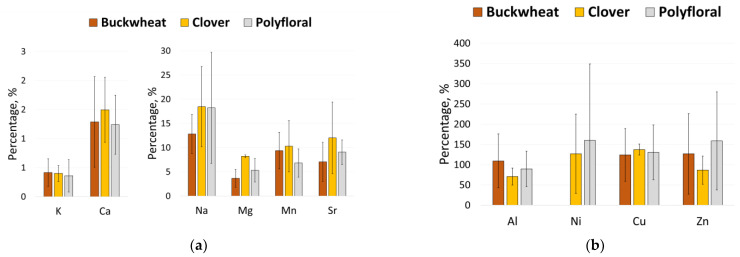
Percentage of element mass concentration in honey proteins compared to the total element concentration in honey: (**a**) elements below the 100% threshold; (**b**) elements exceeding the 100% threshold. Notably, nickel (Ni) was not detected in buckwheat honey when the total Ni concentration was determined.

Interestingly, some honey varieties tend to accumulate a substantial number of proteins, while others do not. In fact, using the proteins gives the representation of honey mass up to 20-fold larger than when using the microwave digestion method. Honeys containing proteins that were both sufficiently abundant and soluble in water were selected for the ICP-TQMS analysis. The mass concentration of elements derived from honey proteins was then compared to the total element mass concentration in honey, and the results are depicted in Figure 3.

The results enabled us to determine whether elements were bound to proteins, existed as free ions, or were associated with smaller molecular compounds (organometallic compounds). The findings indicated that, in honey, the concentrations of Na, Mg, Sr, Mn, Ca, and K within proteins ranged from only 0.10% to 57% of the total element mass concentration. This suggests that these elements in honey are generally present as free ions in solution or are bound to smaller molecular compounds, such as sugars. Zn, Cu, Ni, and Al exhibited nearly 100% of the total element mass concentration, and in some cases, they were even 15-fold greater. This strongly suggests that the largest portion of total elements is bound to proteins. This raises a pertinent question: Are these elements bioavailable from honey? While several studies have touted honey as an excellent source of these nutritional elements [79], it is challenging to find conclusive evidence in the literature. Pohl et al. conducted research on the bioaccessibility of Ca, Cu, Mg, Mn, and Zn in honey and found that the percentage of these elements that is bioaccessible from the total element concentration is remarkably high [80]. Interestingly, our study supports the notion that most Cu and Zn is bound to proteins, likely exceeding 10 kDa in size. Honey has been confirmed to offer various health benefits, including anti-inflammatory properties [81], antioxidative effects [82], antibacterial properties [83], support for gastric and digestive health [84], cancer prevention potential [85], cancer treatment possibilities [86], and benefits for skin care [87]. Additionally, honey contains several other important compounds, such as polyphenols [88], water-soluble vitamins [89], free amino acids [90], and enzymes [91].

However, discerning the specific compounds or classes of compounds responsible for these health benefits is a challenging task that requires critical assessment. The existing literature is relatively limited when it comes to studies investigating the health benefits of protein-bound Zn, Cu, Ni, and Al in honey, or medicinal preparations of plant origin. Exploring this area could be of great interest, given the significance of the health benefits derived from natural ingredients, which holds potential importance for both the field of apiculture and human health. It may seem counterintuitive to find higher element concentrations in honey proteins compared to honey itself, but this phenomenon can be attributed to several factors, including method uncertainties, potential errors, and variations in sample preparation techniques, such as mineralization and simple solvation in HNO_3_ acid.

The use of a straightforward dilution method with HNO_3_ acid solution for sample preparation offered advantages by avoiding additional steps and potential losses, such as the loss of volatile elements during the mineralization phase [92]. Additionally, the total element content in the samples was often near the limit of quantification (LOQ), leading to increased uncertainty in the outcome of the results [93].

Notably, in the protein fractions, Cd and As were not detected. Cr was consistently found in all honey proteins, while in direct honey analysis, most results fell below the limit of detection (LOD). As for Pb, it was present in the majority of protein samples, with estimated concentrations ranging from 0.006 to 0.1 mg/kg. In contrast, only 33 out of 83 honey samples exhibited Pb levels above 0.0002 mg/kg. This variation can be attributed to differences in the affinities of metals for protein binding. For instance, it is possible that elements like Cr and Pb exhibit a strong affinity for the proteins in honey, resulting in their consistent presence in protein fractions. In contrast, elements like Cd and As may not share the same strong binding affinity, making them less detectable in these fractions [94,95,96,97].

### 3.6. Chemometrics

**Figure 4:**
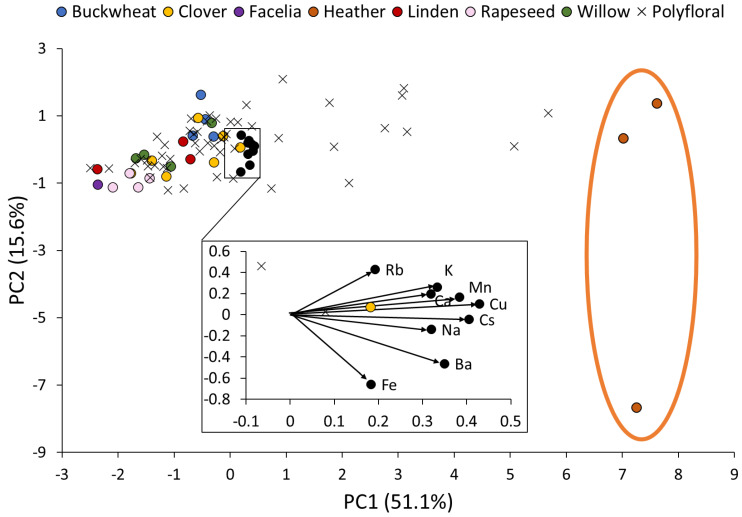
Biplot of principal component score plot (PC1–PC2) and its loading plot. Scores and loadings (showed as black markers) are constructed from the Na, K, Ca, Mn, Fe, Cu, Rb, Cs, and Ba concentration correlation matrix of Latvian honey of different floral origins. Within the orange marker, the separation of heather honey samples from other samples is depicted.

For principal component analysis, nine elements were chosen (Na, K, Ca, Mn, Fe, Cu, Rb, Cs, Ba) because, after the current evaluation, they represented the floral origins most successfully. The biplot of the score plot and loading plot is shown in Figure 4. 

In total, 66.7% of the variance was explained by PC1–2 scores. By using the Mahalanobis distance outlier test, the polyfloral samples that exceeded the critical value (4269) were excluded from further PCA calculations. The monoflorals were kept as reference standards for the floral origin direction. It was predictable that monofloral samples should be far from others because of different physicochemical characteristics. The sample size was reduced from 83 to 76. PC1 was influenced only positively by all elements. Cu and Cs had the most influence, followed by Mn, Ba, K, Na, and Ca element concentrations, but Rb and Fe were comparatively slight. PC1 showed the separation of heather honey from the other samples.

**Figure 5:**
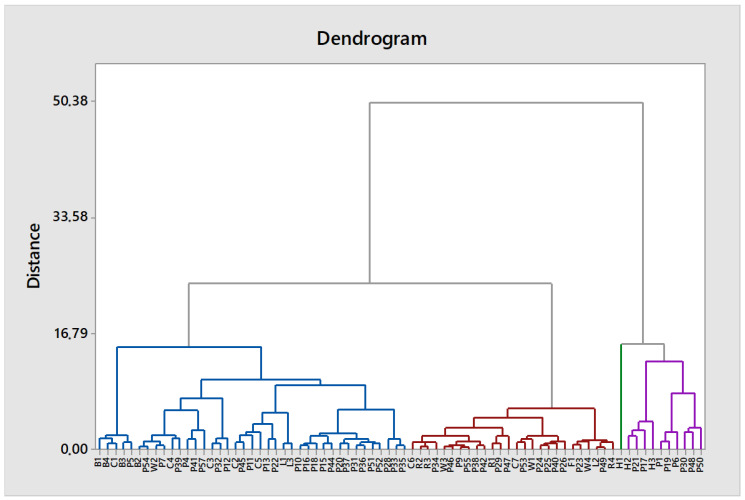
Hierarchical cluster analysis (HCA) dendrogram of Latvian honey of different floral origins. The dendrogram is divided into four groups by Na, K, Ca, Mn, Fe, Cu, Rb, Cs, and Ba element profile similarity: blue, red, green, and purple. Floral origins are denoted with the first letter of code as follows: B—buckwheat, C—clover, H—heather, L—linden, R—rapeseed, W—willow, and P—polyfloral.

HCA was applied to the previous 76-sample dataset without outliers. The HCA dendrogram is shown in Figure 5.

According to Kaufman et al. [98], the optimal number of clusters k is the one that maximizes the average silhouette over a range of possible values for k; thus, in our study the maximum was reached with four groups. The buckwheat honey samples were grouped in the blue group and rapeseed in the red group. The green group consisted only of one sample (H1) and was located next to the purple group, which contained other heather monoflorals and eight polyfloral samples (P1, P6, P17, P19, P21, P30, P48, and P50) with pollen percentage from 0 to 22%. The excluded samples P2, P3, P8, and P14 had relatively high heather pollen percentages (24–38%). Similarity between other monofloral types was inconsistent. The linden honey samples L1 and L3 were grouped in the blue group next to each other, but L2 was in the red group. The clover and willow honey samples were scattered throughout the blue and the red groups. 

Considering only nine variables were used for the PCA and cluster analysis, the separation of monofloral heather honey and other floral origins was observed. The polyflorals with large heather pollen contents were excluded due to different patterns as outliers. The lack of selectivity and ability to determine a threshold for monofloral honey are major issues to using a chemometric approach. The addition of new variables from other methods could be a solution to improve the selectivity of the determination of the most common floral origins of Latvia using chemometric methods.

## 4. Conclusions

The multi-element profile is applicable as a method for evaluating the floral origins of honey. The results of the current study show the possibility of identifying heather honey by evaluating Ca, Fe, Rb, Cs, and Ba concentrations, while the Cu concentration (for buckwheat honey) and Sn concentration (for willow or linden honey) can be valuable secondary indicators of floral origins if a multi-method approach is used. The characterization of the multi-element profile of Latvian polyfloral honey is helpful for distinguishing it from monofloral honey, but concerns are raised when element concentration is near the LOD. Currently, the monofloral group evaluation using Co, Cr, Ni, and Pb is not applicable due to quantification results near the LOD. The As and Tl concentrations could be promising indicators for heather honey, but an increase in sensitivity is required. This suggests that these elements are common in Latvian honey, although in currently undetectable concentrations. The characterized concentrations are in good agreement with those from other studies and indicate that Latvian polyfloral honey is quite rich in minerals in the global context. While the current study emphasized buckwheat, clover, heather, linden, rapeseed, and willow floral origins, the less common plant pollens present in honey were also compared to multi-element concentrations. The results of the Pearson correlation analysis revealed the plausible use of the element profile as a floral origin determination tool for broad bean, umbellifer, and meadowsweet honey. The results of the element profile of honey and its proteins showed that Ca, K, Mg, Mn, Na, and Sr were mainly found in a non-protein-binding form and are thus arguably bioavailable from honey. Al, Cu, Ni, and Zn seem to exist in a protein-binding form with large molecular compounds (>10 kDa); therefore, their bioavailability from honey would seem doubtful. However, in vitro studies suggest a near 100% bioavailability for Cu and Zn. This tempts us to further investigate the honey protein profile. Attention should be paid to differences in the methods of both the honey and protein fraction element determination. Two-stage mineralization allows the use of a larger sample weight and the determination of less common trace elements, but the results might be achieved at the expense of volatile element losses. Conversely, the proteins were prepared by avoiding any extra sample preparation steps, and by using the triple-quadrupole mode, the strong influence of matrix effects was omitted. The honey proteins provided up to 20-fold greater insights into the multi-element profile, thus indirectly achieving a more sensitive determination of whether the majority of the elements were bound with proteins. Cr and Pb were found in honey proteins, while As and Cd were absent. The chemometric evaluation confirmed previous findings describing an outstanding determination of heather honey and polyflorals rich in heather pollen. The low number of monoflorals could impact result validity, but this preliminary study gives better insights and prepares us for future studies to investigate the ability to recognize monofloral honey from polyfloral samples.

## Figures and Tables

**Table 1:** Dominant pollen range and legislation requirements of monofloral samples [34].

Monofloral Type	n	Dominant Pollen Percentage Range, %	Legislation Required Pollen Percentage, %
Buckwheat (*Fagopyrum esculentum)*	4	40–48	>25
Clover (*Trifolium repens*)	7	49–83	>45
Facelia (*Phacelia tanacetifolia*)	1	82	>45
Heather (*Calluna vulgaris*)	3	42–80	>40
Linden (*Tilia cordata*)	3	37–91	>17
Rapeseed (*Brassica napus*)	4	72–89	>70
Willow (*Salix cinerea*)	4	52–66	>45

**Table 2:** Inductively coupled plasma mass spectrometer (ICP-MS) Agilent 7700× parameters for multi-element total determination.

Parameter	Set Value
Plasma mode	Normal, robust
RF forward power	1300 W
Sampling depth	8.0 mm
Plasma gas flow	15.0 L/min
Carrier gas flow	0.6 L/min
Dilution gas flow	0.4 L/min
Spray chamber temperature	2 °C
Extraction lens	0 V
Kinetic energy discrimination	3 V

**Table 3:** Inductively coupled plasma triple-quadrupole mass spectrometer (ICP-TQMS) Agilent 8900 Triple Quadrupole parameters for multi-element determination in honey proteins.

Parameter	Set Value
RF forward power	1550 W
Sampling depth	8.0 mm
Plasma gas flow rate	15.0 L/min
Nebulizer gas flow	0.9 mL/min
He cell gas flow	5.0 mL/min
Extraction lens 1	−17.2 V
Extraction lens 2	−250 V
Omega lens	5.8 V
Omega bias lens	−140 V
Octopole bias	−18 V
Cell gas flow rate	5.0 mL/min

**Table 4:** The validation results of multi-element analysis of carbohydrates using ICP-MS.

Element	LOD, mg/kg	LOQ, mg/kg	Range, mg/kg	Recovery, %	Repeatability, RSD%
Li	0.008	0.025	0.025–50	103	3
Be	0.008	0.025	0.025–50	101	2
Na	0.15	0.5	0.5–5000	91	1
Mg	0.015	0.05	0.05–5000	92	1
Al	0.075	0.25	0.25–50	98	7
K	0.075	0.25	0.25–5000	101	2
Ca	0.75	2.5	2.5–5000	95	3
V	0.008	0.025	0.025–50	108	3
Cr	0.045	0.15	0.15–50	99	6
Mn	0.008	0.025	0.025–50	97	1
Fe	0.075	0.25	0.25–1000	108	4
Co	0.008	0.025	0.025–50	113	3
Ni	0.015	0.05	0.05–50	111	2
Cu	0.045	0.15	0.15–50	103	1
Zn	0.075	0.25	0.25–50	86	6
Ga	0.008	0.025	0.025–50	113	2
As	0.0015	0.005	0.005–2.5	99	6
Se	0.0015	0.005	0.005–2.5	115	10
Rb	0.008	0.025	0.025–50	114	2
Sr	0.008	0.025	0.025–50	112	2
Mo	0.008	0.025	0.025–50	112	2
Ag	0.008	0.025	0.025–50	107	14
Cd	0.002	0.005	0.005–50	103	7
Sn	0.008	0.025	0.025–2.5	109	2
Sb	0.002	0.005	0.005–2.5	101	3
Cs	0.008	0.025	0.025–50	109	2
Ba	0.008	0.025	0.025–50	112	1
Hg	0.0008	0.0025	0.0025–0.05	85	5
Tl	0.008	0.025	0.025–50	103	2
Pb	0.0002	0.0005	0.0005–50	116	8

**Table 5:** Multi-element profile of honey samples from Latvia of different floral origins.

Element	Floral Origins, Pooled Mean ± Standard Deviation, mg/kg						
	Buckwheat (*n* = 4)	Clover (*n* = 7)	Heather (*n* = 3)	Linden (*n* = 3)	Rapeseed (*n* = 4)	Willow (*n* = 4)	Polyfloral (*n* = 57)
Na	7 ± 3 ^B^	11 ± 4 ^AB^	26 ± 5 ^A^	5.1 ± 0.3 ^B^	7.0 ± 1.8 ^B^	7 ± 2 ^B^	13 ± 13 ^AB^
Mg	16 ± 6 ^AB^	11.0 ± 1.9 ^B^	16.397 ± 0.012 ^AB^	13.5 ± 0.9 ^AB^	13.8 ± 1.7 ^AB^	14 ± 4 ^AB^	22 ± 15 ^A^
K	490 ± 100 ^BC^	500 ± 200 ^C^	1500 ± 300 ^A^	1100 ± 700 ^AB^	320 ± 80 ^C^	550 ± 130 ^BC^	900 ± 500 ^B^
Ca	35 ± 8 ^BC^	34 ± 4 ^C^	75 ± 18 ^A^	28.0 ± 1.7 ^C^	39 ± 6 ^BC^	38 ± 8 ^BC^	51 ± 18 ^B^
Mn	6 ± 2 ^AB^	3 ± 2 ^BC^	12 ± 2 ^A^	0.29 ± 0.10 ^C^	0.37 ± 0.14 ^C^	2.1 ± 1.4 ^BC^	4 ± 4 ^BC^
Fe	1.2 ± 0.5 ^B^	0.8 ± 0.4 ^B^	7 ± 7 ^A^	<0.075 ^B^	1.4 ± 0.6 ^B^	<0.075 ^B^	1.4 ± 1.0 ^B^
Cu	0.65 ± 0.16 ^A^	0.3 ± 0.3 ^AB^	0.34 ± 0.06 ^BC^	<0.15 ^C^	<0.15 ^C^	0.27 ± 0.17 ^BC^	0.33 ± 0.22 ^B^
Rb	0.8 ± 0.2 ^B^	1.6 ± 1.4 ^B^	7.7 ± 1.9 ^A^	1.5 ± 0.9 ^B^	0.36 ± 0.17 ^B^	0.8 ± 0.2 ^B^	2 ± 2 ^B^
Sr	0.042 ± 0.016 ^AB^	0.036 ± 0.008 ^B^	0.043 ± 0.010 ^AB^	0.069 ± 0.018 ^AB^	0.07 ± 0.04 ^AB^	0.044 ± 0.005 ^AB^	0.062 ± 0.018 ^A^
Sn	<0.025 ^B^	<0.025 ^B^	<0.025 ^AB^	0.034 ± 0.009 ^AB^	<0.025 ^B^	0.039 ± 0.013 ^AB^	0.039 ± 0.019 ^A^
Cs	<0.025 ^C^	<0.025 ^C^	0.24 ± 0.05 ^A^	<0.025 ^C^	<0.025 ^C^	<0.025 ^C^	0.13 ± 0.07 ^B^
Ba	<0.025 ^B^	0.05 ± 0.02 ^B^	0.6 ± 0.4 ^A^	0.07 ± 0.02 ^B^	<0.025 ^B^	0.041 ± 0.008 ^B^	0.09 ± 0.08 ^B^

^ABC^ results indicated with a different superscript letter are significantly different using the ANOVA one-way Fisher test (*p* < 0.05).

## Data Availability

Data are contained within the article.

## Supplementary Materials

The following supporting information can be downloaded at: https://www.mdpi.com/article/10.3390/foods12224091/s1, Figure S1: Correlation chart between element concentrations and pollen percentage; Tables S1–S5: Multi-element concentration chart (mg/kg) in honey samples using ICP-MS.
