# Supplementary material for: Multi-Element Profile Characterization of Monofloral and Polyfloral Honey from Latvia
Source: Foods. 2023 Nov 11;12(22):4091. doi: 10.3390/foods12224091 (PMC10670016; doi:10.3390/foods12224091)
Supplement: Supplementary file 1 [file foods-12-04091-s001.zip › Supplementary Figure 1.pdf]

	Brassicaceae	*Tilia*	*Ericaceae*	*Fagopyrum esculentum*	*Trifolium spp.*	*Trifolium pratense*	*Rubus*	*Salicaceae*	*Centaure acyanus*	*Vicia faba*	*Phacelia tanacetifolia*	*Apiaceae*	*Betula pendula*	*Taraxacum officinale*	*Prumus/Pyrus*	*Lathyrus spp.*	*Pilipendula ulmaria*	*Matricaria spp.*	*Solidago virgaurea*	*Polygonum bistorta*	*Salvia spp.*	*Echium vulgare*
**Na**	0.109	0.050	0.072	-0.138	-0.070	-0.031	-0.101	-0.078	0.270	0.064	0.025	-0.125	-0.026	-0.066	-0.058	-0.050	0.005	0.121	-0.060	-0.064	-0.018	-0.081
**Mg**	0.036	-0.015	0.032	-0.042	-0.158	-0.065	-0.058	0.028	-0.067	0.009	0.046	0.010	-0.083	-0.086	0.062	0.281	-0.119	-0.024	-0.102	-0.058	-0.050	0.046
**Al**	0.051	-0.022	0.025	-0.129	-0.053	-0.085	0.094	0.201	-0.050	0.026	0.013	0.013	0.259	-0.052	-0.055	0.038	-0.062	0.014	-0.019	-0.063	-0.005	-0.054
**K**	-0.024	0.226	0.135	-0.127	-0.235	-0.135	-0.099	-0.094	-0.056	-0.006	0.079	-0.071	-0.107	0.043	0.176	0.212	-0.143	0.173	-0.134	-0.163	-0.013	0.035
**Ca**	-0.052	0.000	0.157	-0.169	-0.198	0.009	-0.122	-0.021	-0.015	0.130	0.057	0.038	-0.104	-0.047	0.022	0.018	-0.108	0.126	-0.179	-0.103	-0.003	0.048
**Mn**	-0.141	0.007	0.243	0.183	-0.097	-0.136	-0.130	-0.125	0.067	-0.130	0.101	-0.073	-0.053	-0.053	-0.090	0.001	-0.069	0.248	-0.086	-0.087	-0.017	-0.048
**Fe**	-0.126	-0.115	0.579	-0.026	-0.054	0.198	0.084	0.095	-0.127	-0.066	0.133	0.017	-0.014	-0.065	0.002	*	-0.058	*	-0.021	-0.065	*	*
**Co**	0.052	-0.111	-0.068	-0.111	0.030	-0.089	0.422	0.219	-0.064	-0.079	-0.099	-0.112	0.579	-0.048	-0.087	-0.058	*	*	*	*	*	-0.063
**Ni**	0.212	-0.093	-0.058	0.040	0.026	-0.039	0.084	0.137	-0.114	-0.082	-0.076	-0.018	0.381	-0.049	-0.050	-0.023	0.192	-0.058	-0.050	*	0.204	-0.049
**Cu**	-0.207	-0.096	0.069	0.373	0.059	0.002	-0.142	-0.084	-0.135	-0.141	0.054	0.046	-0.146	-0.147	0.012	0.228	0.016	-0.010	-0.108	*	0.054	-0.016
**Zn**	0.134	-0.105	-0.032	-0.092	-0.041	-0.073	-0.035	-0.054	-0.103	-0.073	0.313	-0.065	-0.069	-0.049	0.094	0.108	-0.092	-0.013	-0.042	-0.057	-0.053	-0.032
**Se**	0.636	-0.358	0.308	0.745	-0.273	-0.135	0.326	-0.256	-0.290	-0.290	-0.015	0.066	-0.508	-0.297	*	*	*	0.308	*	*	*	*
**Rb**	-0.069	0.097	0.294	-0.107	-0.139	-0.166	-0.057	-0.136	0.114	-0.108	0.098	-0.115	0.056	0.039	-0.011	0.027	-0.105	0.380	-0.079	-0.091	-0.036	-0.040
**Sr**	0.089	0.018	0.077	-0.147	-0.210	-0.068	-0.019	-0.101	-0.022	0.061	-0.033	-0.016	-0.069	-0.022	0.106	-0.009	-0.109	-0.075	-0.085	-0.059	-0.044	0.013
**Cd**	0.315	0.249	-0.159	0.121	0.357	0.121	0.012	-0.213	-0.260	-0.260	-0.209	0.763	*	*	-0.259	*	*	*	*	*	*	-0.259
**Sn**	0.042	0.115	-0.054	0.022	-0.021	-0.146	-0.079	0.093	-0.173	-0.025	-0.089	-0.310	-0.175	-0.207	0.032	*	-0.123	*	*	-0.016	-0.153	*
**Cs**	0.342	0.326	0.080	-0.150	-0.313	-0.360	-0.016	-0.262	0.571	0.836	-0.111	-0.136	-0.098	-0.146	-0.155	-0.147	*	0.182	*	*	*	*
**Ba**	-0.135	-0.014	0.673	-0.122	-0.137	-0.146	-0.055	-0.098	-0.036	-0.089	0.032	0.004	-0.012	0.034	0.006	-0.029	-0.089	0.283	-0.053	-0.056	-0.052	-0.049
**Tl**	0.615	0.133	-0.115	-0.026	0.027	-0.152	0.082	-0.211	0.426	0.909	-0.200	-0.214	-0.112	-0.204	-0.189	*	*	0.074	*	*	*	*
**Pb**	0.077	-0.125	-0.076	0.308	0.080	-0.020	-0.167	-0.139	-0.147	-0.108	0.405	-0.116	-0.111	-0.087	-0.065	-0.078	0.600	-0.075	*	*	*	*

Figure S1. Correlation chart between element concentrations and pollen percentage. Pollen plants names in Latin. Symbols "*" Means there are no samples with specific pollen over LOD. Color code: green means positive correlation, yellow means weak or no correlation and red means negative correlation.
