# Supplementary material for: Multi-Element Profile Characterization of Monofloral and Polyfloral Honey from Latvia
Source: Foods. 2023 Nov 11;12(22):4091. doi: 10.3390/foods12224091 (PMC10670016; doi:10.3390/foods12224091)
Supplement: Supplementary file 1 [file foods-12-04091-s001.zip › Supplementary Table 1-5.pdf]

Table S1. Multielement concentration chart (mg/kg) in honey samples using ICP-MS. Samples coded B1-4 are buckwheat, C1-7 clover, F1 is Phacelia, H1-3 are heather, L1-3 are linden origins honey samples.

	B1	B2	B3	B4	C1	C2	C3	C4	C5	C6	C7	F1	H1	H2	H3	L1	L2	L3
**Li**	n.d.	n.d.	n.d.	n.d.	<LOD	n.d.	n.d.	n.d.	n.d.	n.d.	<LOD	n.d.	<LOD	n.d.	n.d.	n.d.	n.d.	*0.011*
**Be**	n.d.	n.d.	n.d.	n.d.	n.d.	n.d.	<LOD	n.d.	n.d.	n.d.	n.d.	n.d.	n.d.	n.d.	n.d.	n.d.	n.d.	<LOD
**Na**	2.9	10.2	7.9	5.2	5.6	6.6	18.6	16.4	10.0	11.2	10.2	5.7	19.8	23.9	32.8	4.6	5.3	5.4
**Mg**	10.61	25.44	17.67	9.60	8.88	11.52	11.97	13.41	9.47	8.60	13.24	6.99	16.41	16.39	~~23.76~~	14.22	12.27	13.91
**Al**	*0.14*	0.39	*0.14*	*0.13*	0.25	0.26	*0.17*	0.28	*0.20*	*0.17*	<LOD	0.40	0.40	0.63	0.27	n.d.	*0.08*	*0.19*
**K**	368.4	584.6	600.6	414.1	321.0	794.7	516.4	581.1	919.7	270.7	298.7	263.5	1193.6	1595.9	1808.6	1771.8	140.6	1487.5
**Ca**	26.2	46.8	36.5	29.0	29.2	36.7	37.9	39.5	29.8	33.1	33.7	17.3	57.1	68.5	100.3	25.7	30.0	28.3
**V**	n.d.	<LOD	n.d.	<LOD	<LOD	n.d.	<LOD	<LOD	<LOD	n.d.	<LOD	<LOD	<LOD	n.d.	n.d.	n.d.	n.d.	<LOD
**Cr**	n.d.	<LOD	<LOD	<LOD	<LOD	<LOD	<LOD	<LOD	<LOD	<LOD	<LOD	*0.05*	<LOD	<LOD	<LOD	n.d.	<LOD	<LOD
**Mn**	9.947	3.834	6.051	5.886	7.780	2.825	0.173	3.821	1.088	0.408	1.746	0.386	8.555	12.688	13.844	0.157	0.344	0.382
**Fe**	n.d.	0.7	*0.1*	1.8	0.9	*0.2*	0.6	1.5	0.4	<LOD	0.8	1.0	14.2	n.d.	0.6	n.d.	*0.1*	~~0.7~~
**Co**	<LOD	<LOD	<LOD	<LOD	<LOD	<LOD	<LOD	<LOD	*0.0166*	<LOD	<LOD	<LOD	<LOD	<LOD	<LOD	<LOD	<LOD	<LOD
**Ni**	n.d.	*0.045*	<LOD	<LOD	<LOD	0.096	<LOD	*0.015*	*0.043*	n.d.	<LOD	*0.041*	*0.043*	*0.016*	*0.026*	*0.023*	n.d.	<LOD
**Cu**	0.894	0.471	0.546	0.669	0.782	0.337	*0.145*	0.542	*0.117*	*0.068*	0.235	*0.105*	0.290	0.300	0.418	*0.117*	<LOD	*0.114*
**Zn**	0.33	0.53	0.53	0.53	0.56	0.61	0.41	0.57	0.41	0.41	0.40	0.34	0.32	0.81	0.42	*0.08*	*0.10*	*0.24*
**Ga**	<LOD	n.d.	<LOD	n.d.	n.d.	<LOD	n.d.	n.d.	n.d.	<LOD	n.d.	n.d.	n.d.	n.d.	n.d.	n.d.	n.d.	n.d.
**As**	n.d.	n.d.	n.d.	n.d.	n.d.	n.d.	n.d.	n.d.	n.d.	0.003	n.d.	n.d.	n.d.	*0.004*	*0.005*	n.d.	n.d.	n.d.
**Se**	n.d.	n.d.	n.d.	n.d.	n.d.	n.d.	n.d.	n.d.	0.0174	n.d.	n.d.	n.d.	n.d.	0.0412	n.d.	n.d.	n.d.	n.d.
**Rb**	0.51	0.93	0.93	1.03	0.64	3.10	0.32	1.89	4.14	0.39	0.64	0.48	5.41	10.03	7.80	2.05	0.23	2.22
**Sr**	0.026	0.067	0.044	0.032	*0.021*	0.044	0.089	0.041	0.033	0.032	0.043	0.036	0.056	0.039	0.033	0.085	0.043	0.078
**Mo**	n.d.	<LOD	n.d.	<LOD	<LOD	n.d.	n.d.	<LOD	<LOD	n.d.	n.d.	<LOD	<LOD	<LOD	n.d.	<LOD	n.d.	<LOD
**Ag**	<LOD	n.d.	*0.0122*	<LOD	<LOD	n.d.	n.d.	n.d.	n.d.	n.d.	n.d.	n.d.	<LOD	<LOD	<LOD	<LOD	<LOD	<LOD
**Cd**	n.d.	n.d.	n.d.	n.d.	n.d.	n.d.	n.d.	n.d.	n.d.	n.d.	n.d.	n.d.	n.d.	n.d.	n.d.	n.d.	n.d.	n.d.
**Sn**	n.d.	<LOD	<LOD	~~0.039~~	*0.014*	~~0.111~~	*0.023*	*0.009*	*0.008*	*0.008*	~~0.032~~	<LOD	~~0.026~~	n.d.	<LOD	0.043	0.025	<LOD
**Sb**	n.d.	<LOD	n.d.	n.d.	n.d.	n.d.	n.d.	n.d.	n.d.	n.d.	n.d.	n.d.	n.d.	<LOD	n.d.	<LOD	n.d.	n.d.
**Cs**	<LOD	<LOD	<LOD	<LOD	n.d.	~~0.0306~~	n.d.	<LOD	~~0.0264~~	<LOD	<LOD	<LOD	0.1728	0.2752	0.2826	<LOD	<LOD	<LOD
**Ba**	*0.0125*	~~0.0515~~	0.0266	*0.0204*	<LOD	0.0838	*0.0134*	0.0340	0.0642	0.0295	*0.0147*	*0.0181*	1.0542	0.4243	0.2028	0.0446	*0.0144*	0.0855
**Hg**	n.d.	n.d.	n.d.	n.d.	0.0205	n.d.	n.d.	n.d.	n.d.	n.d.	n.d.	n.d.	n.d.	n.d.	n.d.	n.d.	n.d.	*0.0018*
**Tl**	n.d.	<LOD	n.d.	<LOD	n.d.	0.0445	n.d.	<LOD	*0.0181*	<LOD	<LOD	<LOD	*0.0138*	0.0311	0.0273	n.d.	<LOD	n.d.
**Pb**	n.d.	0.00277	n.d.	0.03947	n.d.	n.d.	n.d.	*0.00026*	n.d.	n.d.	n.d.	n.d.	<LOD	0.00175	n.d.	*0.00045*	n.d.	0.00179

*The strikethrough measurements are outliners according to Dixon r10 criteria to its representative floral group. Measurements in cursive are between LOD and LOQ. “n.d.” means “not detected”.

Table S2. Multielement concentration chart (mg/kg) in honey samples using ICP-MS. Samples coded R1-4 are rapeseed, W1-4 are willow, P1-10 polyfloral origins honey samples.

	R1	R2	R3	R4	W1	W2	W3	W4	P1	P2	P3	P4	P5	P6	P7	P8	P9	P10
Li	n.d.	n.d.	n.d.	n.d.	n.d.	n.d.	n.d.	n.d.	n.d.	n.d.	*0.009*	n.d.	n.d.	n.d.	n.d.	n.d.	n.d.	n.d.
Be	n.d.	n.d.	<LOD	<LOD	n.d.	n.d.	n.d.	n.d.	n.d.	n.d.	n.d.	n.d.	n.d.	n.d.	n.d.	<LOD	n.d.	n.d.
Na	5.4	9.2	8.1	5.2	5.3	10.3	6.7	5.5	18.3	14.6	20.3	15.8	8.5	12.7	10.0	~~95.4~~	7.0	7.5
Mg	16.07	14.25	11.27	13.49	10.28	19.71	13.46	10.83	15.96	45.51	18.09	16.44	16.83	15.81	13.22	21.90	14.16	24.94
Al	0.28	0.41	*0.13*	*0.17*	0.33	0.44	*0.20*	*0.11*	0.41	0.64	1.16	0.34	*0.08*	0.38	0.49	0.36	*0.17*	0.96
K	304.8	438.7	293.7	224.1	456.5	771.8	510.4	439.3	1187.3	1377.9	1456.9	1003.3	604.5	961.9	789.7	1900.3	567.4	660.0
Ca	48.0	39.3	40.7	30.0	34.6	44.9	46.4	27.0	60.7	~~138.1~~	62.1	49.5	25.3	49.5	46.8	55.6	44.4	51.6
V	<LOD	<LOD	n.d.	<LOD	<LOD	n.d.	n.d.	n.d.	n.d.	n.d.	<LOD	<LOD	n.d.	n.d.	n.d.	<LOD	n.d.	n.d.
Cr	<LOD	<LOD	<LOD	<LOD	<LOD	<LOD	<LOD	n.d.	<LOD	<LOD	<LOD	<LOD	<LOD	<LOD	n.d.	<LOD	<LOD	<LOD
Mn	0.225	0.572	0.272	0.413	3.232	3.800	0.491	1.045	5.771	12.746	12.883	0.667	7.436	6.591	2.943	16.594	0.223	1.398
Fe	2.4	1.1	0.8	1.3	~~2.3~~	*0.2*	*0.1*	n.d.	n.d.	n.d.	2.4	3.0	*0.2*	*0.1*	n.d.	0.5	*0.2*	0.7
Co	<LOD	<LOD	*0.0085*	<LOD	*0.0226*	*0.0092*	*0.0134*	<LOD	<LOD	<LOD	<LOD	<LOD	<LOD	<LOD	<LOD	<LOD	<LOD	*0.0150*
Ni	<LOD	0.904	<LOD	<LOD	*0.018*	*0.018*	n.d.	n.d.	*0.015*	*0.017*	*0.019*	*0.025*	n.d.	n.d.	0.804	0.052	n.d.	0.058
Cu	*0.086*	*0.082*	*0.088*	*0.047*	0.345	0.505	0.079	0.160	0.317	0.600	0.385	0.225	0.680	0.231	0.428	0.434	*0.074*	0.261
Zn	0.40	0.43	*0.25*	*0.20*	0.50	0.60	0.30	0.31	0.80	1.36	0.60	0.55	3.07	0.35	*0.17*	0.71	0.29	0.95
Ga	<LOD	<LOD	n.d.	n.d.	n.d.	n.d.	n.d.	n.d.	n.d.	n.d.	n.d.	<LOD	<LOD	n.d.	n.d.	n.d.	n.d.	n.d.
As	n.d.	n.d.	n.d.	n.d.	n.d.	n.d.	n.d.	<LOD	*0.004*	n.d.	n.d.	n.d.	*0.002*	n.d.	n.d.	n.d.	n.d.	n.d.
Se	n.d.	n.d.	n.d.	n.d.	n.d.	n.d.	n.d.	n.d.	n.d.	n.d.	n.d.	n.d.	n.d.	n.d.	n.d.	n.d.	n.d.	n.d.
Rb	0.25	~~0.66~~	0.30	0.23	0.53	0.65	0.84	1.12	3.36	5.67	6.63	1.10	0.86	4.15	1.35	8.72	0.28	1.38
Sr	0.060	0.128	0.036	0.040	0.040	0.048	0.039	0.049	~~0.288~~	0.069	0.045	0.081	0.051	0.047	0.048	0.046	0.055	0.071
Mo	<LOD	*0.016*	<LOD	n.d.	<LOD	n.d.	n.d.	n.d.	n.d.	*0.009*	<LOD	<LOD	n.d.	n.d.	n.d.	n.d.	n.d.	n.d.
Ag	n.d.	n.d.	<LOD	<LOD	<LOD	n.d.	n.d.	n.d.	<LOD	<LOD	n.d.	<LOD	<LOD	<LOD	<LOD	n.d.	n.d.	n.d.
Cd	n.d.	n.d.	n.d.	n.d.	n.d.	n.d.	n.d.	<LOD	n.d.	<LOD	n.d.	n.d.	<LOD	n.d.	n.d.	<LOD	n.d.	<LOD
Sn	<LOD	<LOD	<LOD	n.d.	*0.024*	0.057	0.036	0.025	n.d.	n.d.	0.052	n.d.	0.059	*0.012*	*0.009*	0.033	*0.009*	n.d.
Sb	n.d.	*0.0042*	n.d.	n.d.	n.d.	n.d.	n.d.	n.d.	n.d.	n.d.	<LOD	n.d.	n.d.	n.d.	n.d.	n.d.	n.d.	n.d.
Cs	n.d.	<LOD	n.d.	n.d.	<LOD	<LOD	<LOD	<LOD	0.2022	0.1076	0.0890	<LOD	<LOD	0.0816	<LOD	~~0.7833~~	<LOD	*0.0212*
Ba	*0.0142*	0.0297	<LOD	~~0.0318~~	0.0307	0.0421	*0.0232*	0.0488	0.0995	0.3706	0.3265	0.0450	0.0342	0.2227	*0.0201*	0.0528	0.0283	0.0960
Hg	n.d.	n.d.	0.0193	n.d.	n.d.	n.d.	n.d.	n.d.	n.d.	n.d.	n.d.	n.d.	n.d.	n.d.	n.d.	*0.0011*	n.d.	n.d.
Tl	<LOD	<LOD	<LOD	n.d.	<LOD	n.d.	<LOD	n.d.	*0.0205*	*0.0181*	*0.0178*	n.d.	n.d.	*0.0097*	n.d.	0.0992	n.d.	<LOD
Pb	0.00608	0.00146	n.d.	*0.00038*	0.00109	n.d.	n.d.	n.d.	n.d.	0.00089	*0.00033*	0.00111	n.d.	0.00107	n.d.	n.d.	n.d.	n.d.

*The strikethrough measurements are outliners according to Dixon r10 criteria to its representative floral group. Measurements in cursive are between LOD and LOQ. “n.d.” means “not detected”.

Table S3. Multielement concentration chart (mg/kg) in honey samples using ICP-MS. Samples coded P11-28 polyfloral origins honey samples.

	P11	P12	P13	P14	P15	P16	P17	P18	P19	P20	P21	P22	P23	P24	P25	P26	P27	P28
**Li**	<LOD	<LOD	n.d.	<LOD	n.d.	n.d.	n.d.	n.d.	n.d.	<LOD	n.d.	n.d.	n.d.	n.d.	n.d.	n.d.	n.d.	n.d.
**Be**	<LOD	n.d.	n.d.	n.d.	n.d.	n.d.	n.d.	<LOD	n.d.	n.d.	n.d.	n.d.	n.d.	<LOD	n.d.	n.d.	n.d.	n.d.
**Na**	8.5	24.0	7.6	24.7	7.3	9.1	22.0	9.9	18.7	8.2	19.4	6.7	7.4	9.2	10.1	9.5	27.5	11.7
**Mg**	46.05	13.96	45.27	20.76	31.77	18.90	16.31	22.30	17.67	14.94	26.57	53.44	6.27	16.12	20.09	20.90	18.50	27.61
**Al**	1.10	*0.10*	1.78	0.40	0.30	0.42	0.52	0.29	0.31	*0.15*	0.52	0.80	<LOD	*0.15*	*0.20*	*0.09*	0.42	0.30
**K**	1060.1	686.1	1233.0	1657.8	747.7	731.2	1386.3	709.5	1209.8	1092.0	1482.5	1768.7	374.9	496.6	726.5	519.2	1195.0	965.1
**Ca**	34.4	51.2	37.6	60.5	55.7	51.9	56.4	60.8	60.8	53.1	72.3	50.4	23.8	39.4	38.7	46.8	85.3	81.4
**V**	<LOD	<LOD	n.d.	<LOD	n.d.	<LOD	<LOD	<LOD	n.d.	<LOD	n.d.	n.d.	n.d.	<LOD	<LOD	n.d.	n.d.	n.d.
**Cr**	<LOD	<LOD	<LOD	<LOD	<LOD	<LOD	<LOD	<LOD	<LOD	<LOD	<LOD	n.d.	n.d.	<LOD	<LOD	<LOD	n.d.	n.d.
**Mn**	3.426	0.509	3.168	15.451	3.311	2.326	10.637	2.400	7.587	1.626	10.896	3.611	0.288	0.578	0.500	1.035	0.397	0.737
**Fe**	2.0	0.8	n.d.	0.5	n.d.	1.2	2.6	1.3	*0.1*	1.0	*0.2*	n.d.	n.d.	1.2	0.9	*0.1*	n.d.	n.d.
**Co**	*0.0171*	*0.0094*	*0.0139*	<LOD	<LOD	*0.0100*	<LOD	<LOD	<LOD	<LOD	<LOD	*0.0123*	<LOD	<LOD	<LOD	<LOD	<LOD	*0.0093*
**Ni**	0.132	0.052	0.143	*0.032*	<LOD	0.273	0.121	0.063	<LOD	<LOD	*0.016*	0.121	<LOD	*0.015*	*0.025*	<LOD	n.d.	*0.018*
**Cu**	0.625	*0.130*	0.657	0.371	0.446	0.271	0.406	0.254	0.285	0.232	0.403	0.779	*0.143*	0.166	0.201	0.178	0.468	0.262
**Zn**	2.51	0.33	2.34	0.41	0.87	0.52	0.51	0.93	0.55	0.50	0.68	2.75	~~13.62~~	0.34	0.60	0.55	6.49	0.66
**Ga**	<LOD	n.d.	n.d.	<LOD	<LOD	<LOD	n.d.	n.d.	n.d.	n.d.	n.d.	n.d.	n.d.	n.d.	n.d.	n.d.	n.d.	n.d.
**As**	n.d.	n.d.	n.d.	n.d.	*0.005*	n.d.	n.d.	n.d.	n.d.	n.d.	n.d.	n.d.	<LOD	n.d.	n.d.	n.d.	n.d.	n.d.
**Se**	n.d.	0.0176	n.d.	0.0584	n.d.	n.d.	n.d.	n.d.	n.d.	n.d.	n.d.	n.d.	n.d.	n.d.	n.d.	n.d.	n.d.	n.d.
**Rb**	3.54	0.56	3.02	8.42	1.37	1.80	7.06	2.24	4.69	1.99	8.46	2.68	0.52	0.61	0.64	0.85	0.74	0.91
**Sr**	0.056	0.088	0.061	0.056	0.085	0.061	0.039	0.073	0.038	0.055	0.058	0.058	*0.023*	0.054	0.063	0.064	0.103	0.063
**Mo**	*0.021*	<LOD	*0.013*	<LOD	<LOD	*0.009*	*0.010*	<LOD	n.d.	n.d.	n.d.	0.026	n.d.	n.d.	n.d.	n.d.	<LOD	<LOD
**Ag**	n.d.	n.d.	<LOD	n.d.	<LOD	<LOD	n.d.	<LOD	n.d.	n.d.	<LOD	<LOD	n.d.	<LOD	<LOD	n.d.	n.d.	n.d.
**Cd**	*0.0035*	<LOD	*0.0035*	n.d.	<LOD	<LOD	n.d.	n.d.	<LOD	<LOD	n.d.	<LOD	<LOD	<LOD	<LOD	*0.0021*	n.d.	<LOD
**Sn**	n.d.	<LOD	<LOD	<LOD	n.d.	*0.008*	<LOD	<LOD	<LOD	n.d.	*0.018*	n.d.	n.d.	n.d.	n.d.	<LOD	<LOD	n.d.
**Sb**	n.d.	n.d.	n.d.	n.d.	<LOD	<LOD	*0.0024*	n.d.	n.d.	n.d.	n.d.	n.d.	n.d.	n.d.	n.d.	n.d.	n.d.	n.d.
**Cs**	0.0626	<LOD	*0.0177*	0.1400	<LOD	*0.0122*	0.2458	<LOD	0.1532	<LOD	0.2495	*0.0196*	<LOD	n.d.	n.d.	<LOD	<LOD	<LOD
**Ba**	0.0681	0.0383	0.0607	0.2430	0.0369	0.0765	0.1420	0.0579	0.1707	0.0323	0.2430	0.0478	<LOD	*0.0233*	0.0301	*0.0197*	0.0364	0.0337
**Hg**	n.d.	n.d.	n.d.	n.d.	n.d.	n.d.	n.d.	n.d.	n.d.	n.d.	n.d.	n.d.	n.d.	n.d.	0.0030	n.d.	n.d.	n.d.
**Tl**	<LOD	<LOD	<LOD	*0.0204*	<LOD	<LOD	0.0275	<LOD	*0.0144*	n.d.	*0.0197*	<LOD	n.d.	<LOD	n.d.	n.d.	<LOD	<LOD
**Pb**	0.00258	n.d.	0.00157	n.d.	0.00165	0.01582	0.00153	0.00714	n.d.	n.d.	n.d.	0.00159	0.03475	n.d.	n.d.	n.d.	0.01038	n.d.

*The strikethrough measurements are outliners according to Dixon r10 criteria to its representative floral group. Measurements in cursive are between LOD and LOQ. “n.d.” means “not detected”.

Table S4. Multielement concentration chart (mg/kg) in honey samples using ICP-MS. Samples coded P29-46 polyfloral origins honey samples.

	P29	P30	P31	P32	P33	P34	P35	P36	P37	P38	P39	P40	P41	P42	P43	P44	P45	P46
**Li**	n.d.	n.d.	n.d.	n.d.	n.d.	n.d.	n.d.	n.d.	<LOD	n.d.	n.d.	n.d.	n.d.	n.d.	n.d.	n.d.	<LOD	n.d.
**Be**	n.d.	*0.009*	n.d.	<LOD	n.d.	n.d.	n.d.	n.d.	n.d.	n.d.	n.d.	n.d.	n.d.	n.d.	n.d.	n.d.	n.d.	<LOD
**Na**	5.2	8.8	5.6	22.0	14.8	9.6	14.3	6.2	10.4	9.1	15.4	8.5	21.3	8.9	4.5	4.9	7.2	6.7
**Mg**	12.84	40.39	18.74	8.84	15.28	13.54	17.67	15.36	13.43	16.46	18.59	17.32	15.21	12.52	10.87	15.93	18.17	17.30
**Al**	0.38	0.41	0.85	*0.14*	0.52	n.d.	*0.19*	<LOD	*0.11*	0.38	0.77	*0.10*	5.22	*0.20*	0.26	n.d.	*0.18*	1.40
**K**	615.7	1985.8	950.4	304.9	1285.2	227.7	1147.6	777.2	926.6	622.7	919.2	673.3	699.0	672.9	353.5	487.3	1093.2	556.0
**Ca**	44.2	83.1	48.8	30.4	70.1	48.1	64.4	53.1	48.2	36.4	39.8	31.6	48.9	43.9	19.9	56.1	40.2	44.8
**V**	<LOD	<LOD	n.d.	<LOD	n.d.	n.d.	n.d.	n.d.	<LOD	<LOD	n.d.	<LOD	<LOD	<LOD	<LOD	n.d.	<LOD	<LOD
**Cr**	<LOD	<LOD	<LOD	<LOD	<LOD	<LOD	n.d.	<LOD	<LOD	<LOD	<LOD	<LOD	*0.07*	<LOD	<LOD	n.d.	<LOD	<LOD
**Mn**	0.387	1.386	1.833	0.368	0.512	0.409	0.519	2.503	0.670	0.901	1.158	1.441	2.929	0.863	11.680	3.277	1.886	0.571
**Fe**	1.7	1.2	n.d.	0.6	*0.1*	*0.1*	n.d.	*0.1*	0.4	0.5	0.3	0.8	3.6	0.9	1.1	n.d.	0.8	0.7
**Co**	<LOD	*0.0247*	<LOD	<LOD	<LOD	<LOD	<LOD	<LOD	*0.0097*	*0.0217*	*0.0107*	<LOD	0.4856	*0.0205*	<LOD	<LOD	<LOD	<LOD
**Ni**	0.149	0.063	n.d.	0.290	*0.026*	n.d.	n.d.	n.d.	<LOD	*0.041*	*0.041*	<LOD	2.765	1.105	<LOD	0.053	<LOD	<LOD
**Cu**	*0.138*	0.443	0.163	*0.065*	0.259	*0.047*	0.182	0.233	0.152	*0.127*	0.363	0.193	0.354	*0.112*	1.056	0.353	0.358	*0.130*
**Zn**	0.48	1.71	1.39	*0.20*	0.38	*0.17*	0.32	0.53	0.51	0.43	2.69	0.45	0.58	0.54	0.60	0.40	0.67	0.42
**Ga**	n.d.	n.d.	<LOD	n.d.	n.d.	n.d.	n.d.	n.d.	n.d.	<LOD	<LOD	<LOD	<LOD	n.d.	n.d.	n.d.	n.d.	n.d.
**As**	n.d.	n.d.	n.d.	n.d.	n.d.	n.d.	<LOD	n.d.	n.d.	n.d.	n.d.	n.d.	n.d.	n.d.	n.d.	n.d.	n.d.	n.d.
**Se**	n.d.	n.d.	n.d.	n.d.	n.d.	n.d.	n.d.	n.d.	n.d.	n.d.	n.d.	n.d.	0.0141	n.d.	n.d.	n.d.	n.d.	n.d.
**Rb**	0.47	3.59	1.88	0.53	1.18	0.16	0.72	1.49	1.74	1.60	0.78	1.21	1.53	0.86	1.15	0.66	2.82	0.45
**Sr**	0.060	0.099	0.067	0.040	0.094	0.074	0.090	0.053	0.052	0.088	0.052	0.044	0.056	0.070	0.027	0.045	0.067	0.070
**Mo**	<LOD	n.d.	n.d.	<LOD	n.d.	n.d.	<LOD	n.d.	n.d.	<LOD	*0.009*	n.d.	<LOD	n.d.	n.d.	n.d.	<LOD	<LOD
**Ag**	<LOD	<LOD	n.d.	<LOD	n.d.	n.d.	n.d.	n.d.	n.d.	n.d.	<LOD	n.d.	n.d.	n.d.	<LOD	n.d.	<LOD	<LOD
**Cd**	n.d.	0.0071	<LOD	<LOD	n.d.	n.d.	<LOD	n.d.	n.d.	n.d.	<LOD	n.d.	n.d.	n.d.	n.d.	n.d.	n.d.	n.d.
**Sn**	<LOD	<LOD	n.d.	n.d.	*0.011*	*0.010*	n.d.	<LOD	<LOD	<LOD	<LOD	n.d.	<LOD	*0.012*	n.d.	n.d.	0.040	*0.023*
**Sb**	n.d.	n.d.	n.d.	n.d.	n.d.	n.d.	n.d.	n.d.	n.d.	n.d.	n.d.	n.d.	0.0116	n.d.	n.d.	n.d.	n.d.	n.d.
**Cs**	<LOD	<LOD	<LOD	<LOD	<LOD	<LOD	<LOD	<LOD	<LOD	<LOD	<LOD	<LOD	*0.0105*	<LOD	n.d.	<LOD	<LOD	<LOD
**Ba**	0.0332	0.0785	0.0314	0.0349	0.0580	*0.0131*	0.0364	0.0350	*0.0153*	0.0312	0.0477	*0.0139*	0.0764	0.0332	*0.0139*	0.0622	0.0649	0.0287
**Hg**	n.d.	n.d.	n.d.	0.0027	n.d.	n.d.	n.d.	n.d.	n.d.	n.d.	n.d.	n.d.	n.d.	<LOD	n.d.	n.d.	n.d.	n.d.
**Tl**	<LOD	<LOD	<LOD	<LOD	n.d.	<LOD	<LOD	<LOD	n.d.	<LOD	<LOD	n.d.	<LOD	n.d.	<LOD	<LOD	<LOD	<LOD
**Pb**	0.00088	0.00079	n.d.	n.d.	n.d.	n.d.	n.d.	n.d.	n.d.	n.d.	0.01103	n.d.	0.00279	n.d.	n.d.	n.d.	n.d.	*0.00023*

*The strikethrough measurements are outliners according to Dixon r10 criteria to its representative floral group. Measurements in cursive are between LOD and LOQ. “n.d.” means “not detected”.

Figure S5. Multielement concentration chart (mg/kg) in honey samples using ICP-MS. Samples coded P47-57 polyfloral origins honey samples.

	P47	P48	P49	P50	P51	P52	P53	P54	P55	P56	P57
**Li**	n.d.	n.d.	n.d.	n.d.	n.d.	n.d.	n.d.	<LOD	n.d.	n.d.	n.d.
**Be**	n.d.	n.d.	n.d.	n.d.	n.d.	n.d.	n.d.	n.d.	n.d.	n.d.	n.d.
**Na**	5.8	6.9	3.4	9.6	3.6	6.7	6.1	10.6	8.0	6.4	15.2
**Mg**	15.23	79.40	12.33	89.33	24.91	19.86	13.20	14.29	17.30	11.26	15.84
**Al**	0.36	2.65	<LOD	4.51	*0.14*	0.34	0.38	0.36	*0.10*	<LOD	0.62
**K**	1113.8	2018.1	163.3	1908.6	1000.6	1059.4	391.6	573.4	616.9	275.3	955.8
**Ca**	38.5	70.2	28.0	67.6	55.5	57.7	37.2	42.3	40.7	28.2	52.5
**V**	<LOD	n.d.	n.d.	<LOD	n.d.	n.d.	n.d.	<LOD	n.d.	<LOD	<LOD
**Cr**	<LOD	<LOD	n.d.	<LOD	<LOD	<LOD	n.d.	<LOD	<LOD	<LOD	<LOD
**Mn**	0.340	5.649	0.302	6.830	1.792	2.520	3.119	4.241	0.372	4.025	7.493
**Fe**	2.5	0.6	n.d.	2.4	n.d.	*0.2*	n.d.	1.3	*0.1*	~~14.3~~	4.4
**Co**	<LOD	*0.0232*	<LOD	0.0394	*0.0099*	<LOD	<LOD	<LOD	<LOD	<LOD	<LOD
**Ni**	<LOD	0.119	<LOD	0.381	*0.043*	0.310	n.d.	0.205	<LOD	*0.019*	*0.047*
**Cu**	*0.107*	0.553	*0.101*	1.066	0.288	0.340	0.325	0.436	*0.147*	0.422	0.332
**Zn**	1.17	2.44	<LOD	3.63	0.51	0.72	0.36	0.67	0.51	0.36	5.86
**Ga**	n.d.	n.d.	n.d.	n.d.	n.d.	<LOD	n.d.	n.d.	<LOD	<LOD	n.d.
**As**	n.d.	n.d.	n.d.	n.d.	n.d.	n.d.	n.d.	n.d.	n.d.	n.d.	n.d.
**Se**	n.d.	0.0398	n.d.	n.d.	n.d.	n.d.	n.d.	0.0149	n.d.	n.d.	n.d.
**Rb**	0.83	6.50	0.30	4.53	1.28	1.88	0.76	1.16	0.61	0.37	3.53
**Sr**	0.093	0.064	0.064	0.098	0.065	0.075	0.043	0.051	0.046	0.056	0.053
**Mo**	n.d.	<LOD	n.d.	<LOD	n.d.	n.d.	n.d.	*0.008*	n.d.	n.d.	<LOD
**Ag**	*0.00891*	<LOD	n.d.	<LOD	n.d.	n.d.	n.d.	n.d.	n.d.	n.d.	n.d.
**Cd**	n.d.	*0.0028*	n.d.	0.0200	*0.0021*	n.d.	<LOD	n.d.	n.d.	n.d.	n.d.
**Sn**	n.d.	n.d.	n.d.	<LOD	n.d.	*0.010*	<LOD	0.025	0.028	n.d.	<LOD
**Sb**	n.d.	n.d.	n.d.	n.d.	n.d.	n.d.	n.d.	*0.0036*	n.d.	n.d.	<LOD
**Cs**	n.d.	0.0292	<LOD	*0.0092*	<LOD	<LOD	<LOD	<LOD	<LOD	<LOD	0.0541
**Ba**	0.0995	0.2069	*0.0168*	0.1633	*0.0233*	0.0706	*0.0166*	0.0399	0.0337	0.0288	0.1220
**Hg**	n.d.	n.d.	n.d.	n.d.	n.d.	n.d.	n.d.	n.d.	n.d.	n.d.	n.d.
**Tl**	n.d.	*0.0086*	<LOD	<LOD	<LOD	n.d.	<LOD	<LOD	<LOD	<LOD	*0.0107*
**Pb**	n.d.	0.00361	n.d.	0.00572	n.d.	0.00100	n.d.	0.00357	n.d.	n.d.	0.03068

*The strikethrough measurements are outliners according to Dixon r10 criteria to its representative floral group. Measurements in cursive are between LOD and LOQ. “n.d.” means “not detected”.
